# Determination of the Main Nucleosides and Nucleobases in Natural and Cultured *Ophiocordyceps* *xuefengensis*

**DOI:** 10.3390/molecules22091530

**Published:** 2017-09-11

**Authors:** Juan Zou, Ling Wu, Zheng-Mi He, Ping Zhang, Zuo-Hong Chen

**Affiliations:** 1 College of Life Science, Hunan Normal University, Changsha 410081, China; zoujuantone@126.com (J.Z.); wuling15963@163.com (L.W.); hezhengmi1101@163.com (Z.-M.H.); zhangping0000@163.net (P.Z.); 2 Key Laboratory of Research and Utilization of Ethnomedicinal Plant Resources of Hunan Province, College of Biological and Food Engineering, Huaihua University, Huaihua 418000, China

**Keywords:** *Ophiocordyceps xuefengensis*, *Phassus nodus*, stroma, nucleosides

## Abstract

*Ophiocordyceps* *xuefengensis*, a recently described species of *Ophiocordyceps* that is associated with the larvae of *Phassus* *nodus* (Hepialidae) in the living root or trunk of the medicinal plant *Clerodendrum* *cyrtophyllum*, is the largest known *Cordyceps* species and is recognized as a desirable alternative for natural *Ophiocordyceps* *sinensis*. This study investigated the main nucleosides and nucleobases in natural and cultured *Ophiocordyceps* *xuefengensis*. The contents of the nucleosides and nucleobases in the natural and cultured samples were determined by reverse phase HPLC. The highest concentration of adenosine was found in the natural fruit body and the cultured stroma, with almost no adenosine in the cadaver of *Phassus* *nodus*. The contents of adenine, guanosine, uridine and uracil in the cultured mycelium were significantly higher than those in the natural sample. Inosine was only detected in the natural samples. Thymidine and 2-deoxyadenosine were only found in the cadaver of *Phassus* *nodus*. Cordycepin was not detected in the five samples examined. These results suggested that the cultured mycelium and cultured stroma of *Ophiocordyceps* *xuefengensis* might be a promising substitute for natural *O. xuefengensis*.

## 1. Introduction

Nucleosides, as pharmaceutical intermediates and/or prodrugs, play an important role in drug development, and as chemotherapeutic agents, they are widely introduced for the medical treatment of cancer [1,2]. Nucleosides and their nucleobases are one of the major active components of *Cordyceps* [3,4]. Up to now, approximately twenty varieties of nucleosides and their analogues, e.g., cordycepin, adenosine, uridine, guanosine, and inosine, have been found in *Cordyceps* [2]. Among these, Cordycepin, namely, 3′-deoxyadenosine, is the most notable adenosine analogue from some *Cordyceps*, showing various pharmacological activities, such as antimicrobial, antitumour, anti-inflammatory and immunomodulation activities [5,6,7]. Adenosine plays an important role in biochemical processes in organisms, is a major nucleoside in *Cordyceps* and is recognized as a chemical marker for the quality control of *Cordyceps sinensis* (*Ophiocordyceps sinensis*) and its substitutes in China [8]. Therefore, the determination of the content and types of nucleosides is essential for pharmacological studies and the quality control of *Cordyceps* and its products.

*Ophiocordyceps xuefengensis* Ting C. Wen et al. a recently described species of *Ophiocordyceps* parasitized with the larvae of *Phassus nodus* (Hepialidae) in the living root or trunk of the medicinal plant *Clerodendrum cyrtophyllum* in the Hunan Province of Southern China, and it is recognized as a desirable alternative for natural *Ophiocordyceps sinensis* [9]. A recent report shows that *O. xuefengensis* enhances the anti-tumour activity of DC-CIK cells, mainly by promoting proliferation [10].

Recently, the anamorphic state of *Ophiocordyceps xuefengensis* was isolated from the stroma of the fungus, and the fungus bearing stroma was cultivated successfully in artificial media [11], which provided us with a good opportunity to systematically analyse the nucleoside content of natural and cultured *O*. *xuefengensis*. In this study, the natural *O*. *xuefengensis*, cultured mycelium, coremium and stroma of *O*. *xuefengensis*, were systematically analysed for seven nucleosides (cordycepin, adenosine, guanosine, uridine, inosine, thymidine and 2-deoxyadenosine) and two nucleobases (adenine and uracil) by high-performance liquid chromatography coupled with high-resolution mass spectrometry.

## 2. Results and Discussion

### 2.1. Calibration Curves, Limit of Detection (LOD), Limit of Quantification (LOQ) and Method Validation

**Figure 1:**
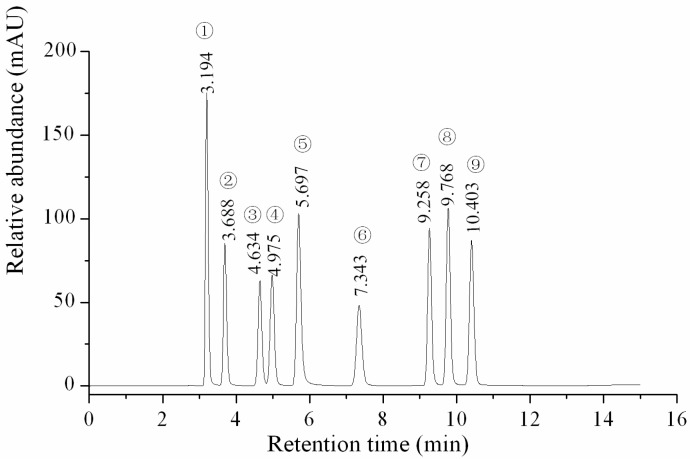
HPLC profile of the standard samples. ① Uracil; ② Uridine; ③ Inosine; ④ Guanosine; ⑤ Adenine; ⑥ Thymidine; ⑦ Adenosine; ⑧ 2-deoxyadenosine; ⑨ Cordycepin.

The linearity, regression, reproducibility and recovery studies and the HPLC profile of nine investigated nucleosides and nucleobases were performed using reference standard samples and are shown in Table 1 and Table 2 and Figure 1. The linearity of each standard was confirmed by plotting the peak area ratio of the individual standard versus the concentration of the standard. The correlation coefficients of all the target components exceeded 0.999, with a good linearity. The LOD and LOQ of the nine analytes were determined at signal-to-noise ratios (S/N) of 3 and 10, and the ranges were 0.002–0.01 and 0.01–0.1 µg/mL, respectively. These data indicated that the HPLC analysis of the nucleosides and nucleobases was sensitive for the qualitative and quantitative determination of the components cordycepin, 2-deoxyadenosine, thymidine, adenosine, guanosine, uridine, inosine, adenine and uracil.

The retention time repeatability of nine nucleosides and nucleobases were tested on seven consecutive days. The result showed that the recoveries were 96.9%, 100.6%, 79.4%, 80.6%, 102.7%, 103.1%, 80.6%, 101.5%, and 90.4% for uracil, uridine, inosine, guanosine, Thymidine, adenosine, adenine, 2-deoxyadenosine and cordycepin, respectively.

### 2.2. Contents of the Nucleosides and Nucleobases in Different Tissues

**Figure 2:**
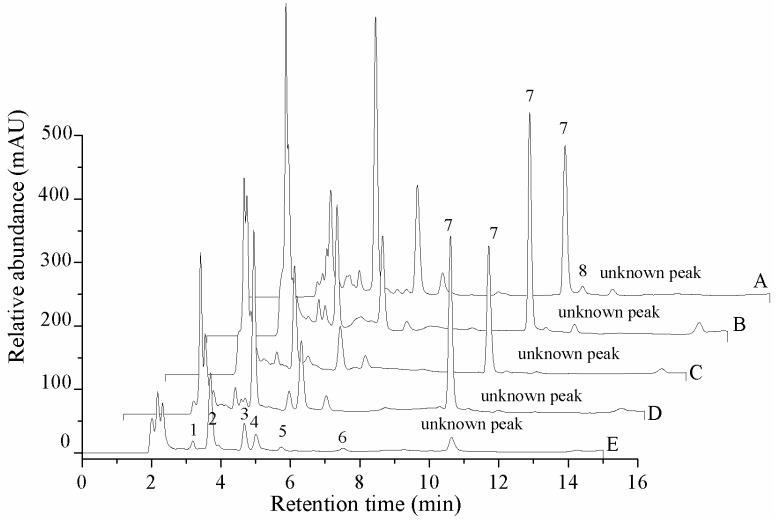
HPLC profile of the extraction of the nucleosides and nucleobases of *O. xuefengensis*. A. Cultured mycelium; B. Cultured stroma; C. Cultured coremium; D. Natural fruit body; E. Cadaver of *phassus nodus*. 1. Uracil; 2. Uridine; 3. Inosine; 4. Guanosine; 5. Adenine; 6. Thymidine; 7. Adenosine; 8. 2-deoxyadenosine.

The contents of seven nucleosides (cordycepin, adenosine, guanosine, uridine, inosine, thymidine and 2-deoxyadenosine) and two nucleobases (adenine and uracil) in five different materials are shown in Figure 2 and Table 3. Adenosine was not detected in the cadaver of *Phassus nodus*, and the content of adenosine in the natural fruit body (761.5 ± 56.4 mg/kg) and cultured stroma (721.2 ± 31.8 mg/kg) was significantly higher than in the cultured mycelium (623.9 ± 15.4 mg/kg) and cultured coremium (582.9 ± 42.6 mg/kg). The relative concentrations of uracil, uridine, inosine, guanosine, adenine, thymidine and 2-deoxyadenosine in the different tissues were compared. Thymidine and 2-deoxyadenosine only were found in the cadaver of *Phassus nodus* and the cultured mycelium, respectively. Inosine only was detected in the natural fruit body and the cadaver of *Phassus nodus*. The content of adenine in the cultured coremium was 186.6 ± 16.6 mg/kg, which was much higher than the other four samples. The highest concentrations of guanosine and uridine were found in the cultured mycelium, which contained 688.9 ± 14.8 mg/kg and 1396.6 ± 49.1 mg/kg, respectively. The content of uracil in the cultured stroma was 138.1 ± 4.8 mg/kg, which was slightly higher than the other four materials. With the exception of the adenosine content, the cultured mycelium contained amounts of adenine, guanosine, uridine and uracil that were significantly higher than the values in the natural *O. xuefengensis*. The contents of adenine, guanosine and uracil in the cultured stroma were also much higher than in the natural *O. xuefengensis*. Therefore, the cultured *O. xuefengensis* mycelium and stroma are possibly helpful to further research on pharmacological activity of *O. xuefengensis*, and might have a promising application in health and functional product development.

The contents of adenosine in the natural stroma and cultured mycelium, stroma, and coremium of *O. xuefengensis* were 760, 620, 720 and 580 mg/kg, respectively, which was slightly lower than natural *O. sinensis* (680−960 mg/kg) reported by Zhao et al. and Wang et al. [12,13], and was significantly higher than *O. sinensis* (46.4−480.2 mg/kg), as reported by Xie et al*.* Zong et al. and Fan et al. [14,15,16]. The adenine content in the three cultured samples, were 126.1, 155.9 and 186.6 mg/kg, respectively, which were higher than those measured for natural *O. sinensis* (4.4−120 mg/kg) [12,13,14,15,16]. There were no significant difference in content of uridine between *O. xuefengensis* and natural *O. sinensis*. The content of guanosine in both the natural and cultured *O. xuefengensis* (179.7−688.9 mg/kg) was lower than the value (1011–1670 mg/g) reported in natural *O. sinensis* by Wang et al*.* and Zong et al*.* [15,17]. Three cultured samples of *O. xuefengensis* and natural *O. sinensis,* reported by Zong et al*.* [17], displayed roughly the same amounts of guanosine, which were 502.1−688.9 mg/kg and 484−832 mg/kg, respectively. These data illustrated that the cultured *O. xuefengensis* mycelium and stroma might be a promising substitute for natural *O. sinensis*.

### 2.3. Identification of Cordycepin

**Figure 3:**
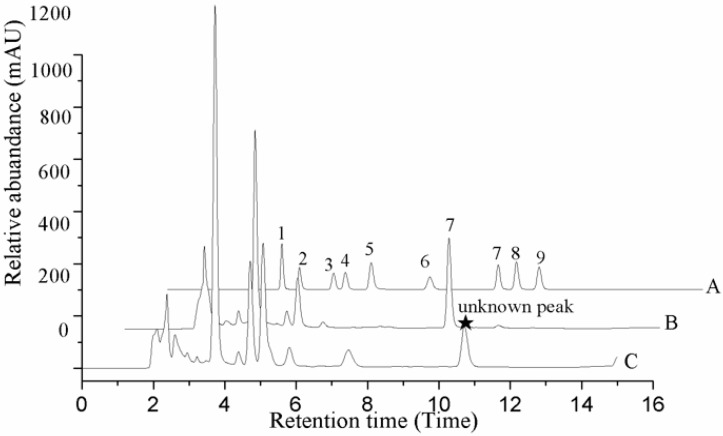
HPLC profile of *O. sinensis*. A. Standard samples. B. Natural fruit body. C. Cadaver of *Hepialidae*. 1. Uracil; 2. Uridine; 3. Inosine; 4. Guanosine; 5. Adenine; 6. Thymidine; 7. Adenosine; 8. 2-deoxyadenosine; 9. Cordycepin.

**Figure 4:**
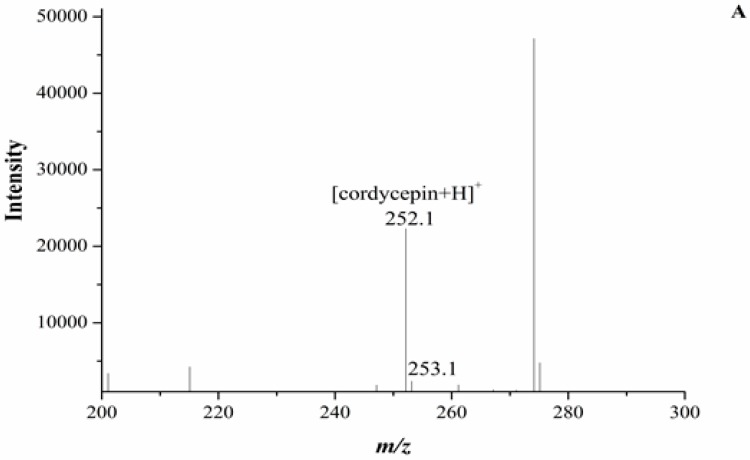

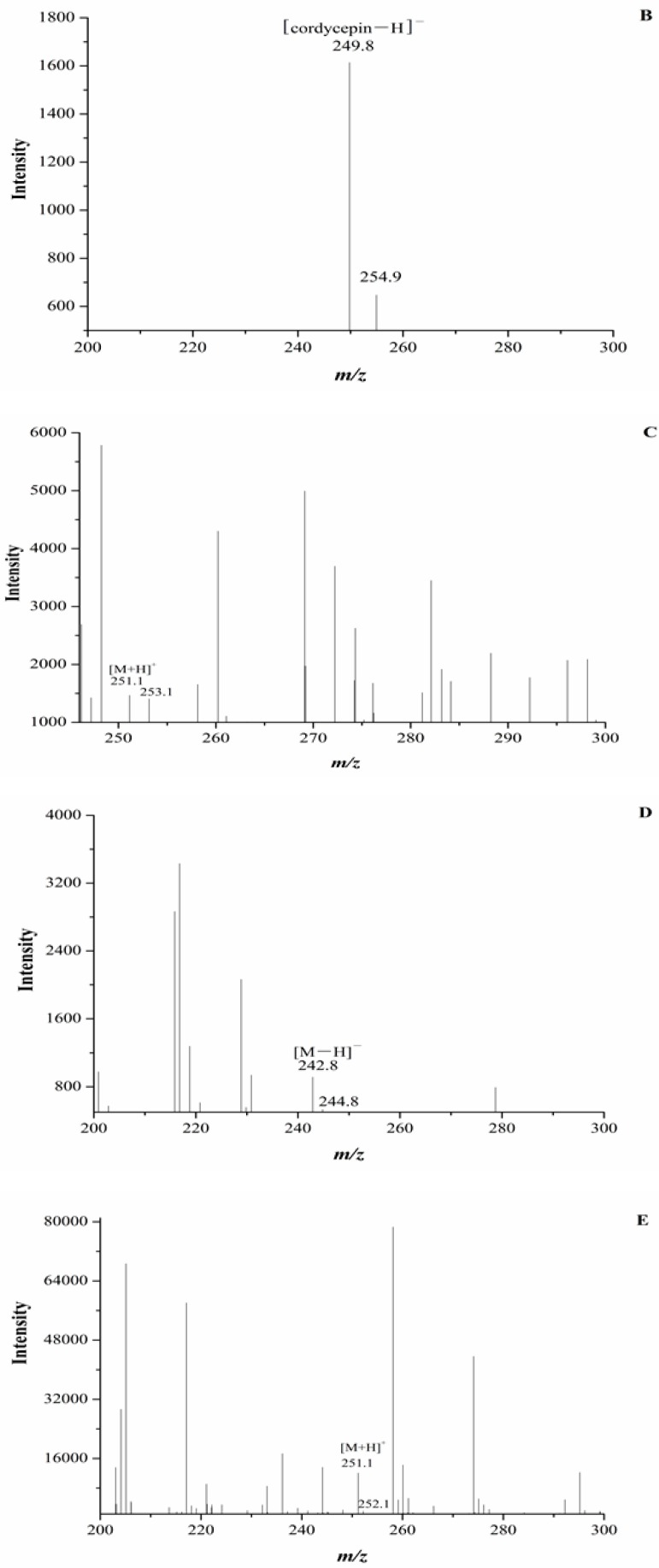

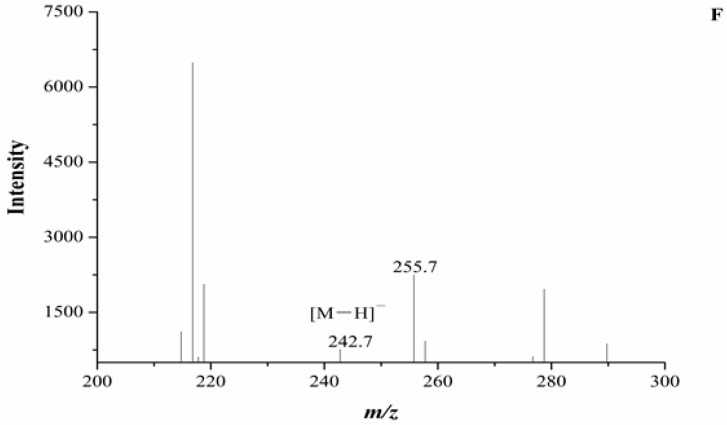
ESI/TOF-MS spectra of cordycepin, *O*. *xuefengensis* and *O. sinensis* in positive and negative ion modes. (**A**) Cordycepin in positive ion mode; (**B**) Cordycepin in negative ion mode; (**C**) The LC peaks *O*. *xuefengensis* in positive ion mode; (**D**) The LC peaks *O*. *xuefengensis* in negative ion mode; (**E**) The LC peaks of *O. sinensis* in positive ion mode; (**F**) The LC peaks of *O. sinensis* in negative ion mode.

In our experiment, the chromatograms of the natural and cultured samples all showed peaks at 10.67 ± 0.05 min after the expected retention times for cordycepin (10.43 ± 0.08 min), and the same peaks at 10.69 ± 0.04 min were found in natural *O. sinensis* (Figure 3). To verify whether *O. xuefengensis* contained cordycepin, water extracts of *O. xuefengensis* and *O. sinensis* were analysed with ESI/TOF-MS. The mass spectrum of cordycepin showed the main peak at *m*/*z* = 252.1, corresponding to the protonated molecular ion, and a peak at *m*/*z* = 249.8, corresponding to the deprotonation molecular ion (Figure 4A,B). However, as Figure 4C,D display, the mass spectrum of the LC peak at 10.67 ± 0.05 min of *O. xuefengensis* had no signal at *m*/*z* = 252.1 in positive mode and no signal at *m*/*z* = 249.8 in negative ion mode, which demonstrated that the LC peak at 10.67 ± 0.05 min of *O. xuefengensis* was not the absorbance peak of cordycepin. The mass spectrum of the LC peak at 10.69 ± 0.04 min of natural *O. sinensis* is shown in Figure 4E, with weak signal at *m*/*z* = 252.1, indicating the protonated species, but no signal was detected at *m*/*z* = 249.8 in negative ion mode (Figure 4F). This may be the reason for the controversy over whether *O. sinensis* contained cordycepin [3,15,18]. In our experiment, cordycepin was not detected in either *O. sinensis* or *O. xuefengensis*. However, cordycepin in the natural *C. sinensis* is confirmed in other reports. In addition, the same unknown LC peaks were also reported in *Cordyceps gunnni* and *C. taii* [2,18], which is likely to be a feature peak in *Ophiocordyceps*, and the identification of the unknown peak requires further research.

## 3. Experimental

### 3.1. Materials

**Figure 5:**
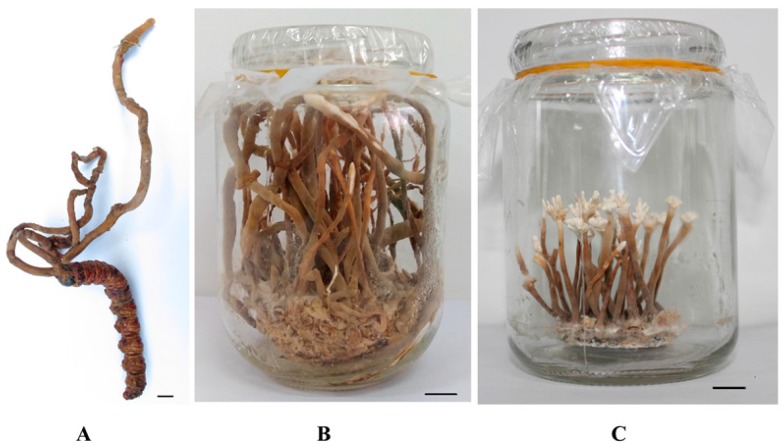
*Ophiocordyceps xuefengensis*. (**A**) Natural *O. xuefengensis*; (**B**) Cultured stroma; (**C**) Cultured coremium. Scale bars: 10 mm.

The fresh stroma of natural *O*. *xuefengensis* (Figure 5A), named MHHNU7966-WS (GenBank accession number KY454625), was collected from *Phassus nodus* in the living root of *Clerodendrum cyrtophyllum* in the Xuefeng Mountains of the Hunan Province in South China. The fungal strain, named MHHNU7966-IS (GenBank accession number KU356800), was isolated from the fresh stroma of natural *O*. *xuefengensis*. The fungus bearing stroma of *O*. *xuefengensis* (Figure 5B) was cultivated in artificial media and collected. The dried fruiting bodies of natural *O. sinensis* were purchased from Qinghai Spring Medicinal Resources Technology Co., Ltd., Xining, Qinghai, China.

### 3.2. Inoculum Preparation and Flask Cultures

The medium for agar slant consisted of the following (g/L): potato infusion, 200; glucose, 10; peptone, 10; pupa powder, 10; and agar, 20.0. The production medium contained the following (g/L): potato infusion, 200; sucrose,15; peptone, 10; Na_2_HPO_4_, 1.18; KH_2_PO_4_, 1.13; MgSO_4_·7H_2_O, 3; KNO_3_, 3; Vitamin B_1_, 0.006; Vitamin B_2_, 0.003; Vitamin B_6_, 0.0004; and nicotinamide, 0.02. The pH values of the media were all adjusted to 6.5 by HCl or NaOH. For the seed preparation, the mycelium of strain MHHNU7966-IS was picked from an agar slant culture, placed into 50 mL of sterile seed medium in a 250 mL Erlenmeyer flask, and incubated at 25 °C with shaking at 150 rpm for 5 days. The production medium in a 250 mL flask was inoculated with 5% (*v*/*v*) of the seed culture and was incubated at 25 °C and 140 rpm for 7 days. The mycelium was collected by centrifugation at 6000×*g* for 15 min.

### 3.3. Artificial Cultivation of Coremium

The solid medium consisting of the following (g/bottle): rice, 10; oat, 5; millet, 2.5; pupa powder, 0.5; CaCO_3_, 0.1; and CaSO_4_·2H_2_O, 0.1, as well as the nutrient solution (30 mL), which contained the following (g/L): potato infusion, 200; sugar,10; peptone, 5; Na_2_HPO_4_, 1.18; KH_2_PO_4_, 1.13; MgSO_4_·7H_2_O, 1.5; and KNO_3_, 2, were poured into a 350 mL glass jar, mixed with slow oscillations, incubated at 50 °C for 2 h, and then sterilized in high-pressure steam at 121 °C for 40 min. The sterilized solid medium in a 350-mL glass jar was inoculated with 2 mL of the seed culture and was incubated for 30 d at 25 °C at 60% relative humidity, in complete darkness. The solid medium, completely covered by mycelium, was used as coremium development. For this the solid medium was incubated with the normal day-night cycle (light intensity was 100–200 lx during the day) at 28 °C for 60 d. The coremium of *O*. *xuefengensis* was obtained in artificial media (Figure 5C).

### 3.4. Extraction of the Nucleoside Analogues and Nucleobases

All the samples were lyophilized to a constant weight and were ground by hand with a mortar and pestle. Samples (0.2 g) of the ground materials (two different natural tissues and three cultured development stages) were weighed accurately into individual test tubes and were extracted with 2 mL of 15% methanol (prepared with doubly distilled water) at 4 °C for 12 h. Following ultrasonic extraction for 30 min and, then, centrifugation at 4000× *g* for 15 min, the supernatant was decanted and retained, and the residue was resuspended in 2 mL of 15% methanol and extracted again as described above. The two supernatants were combined and filtered through a micropore filter membrane (0.22 µm) for the high-performance liquid chromatography (HPLC) analysis. The extraction was donein triplicate for each tissue sample, and the mean value was determined.

### 3.5. Standard Samples and the Linear Regression Equation

**Figure 6:**
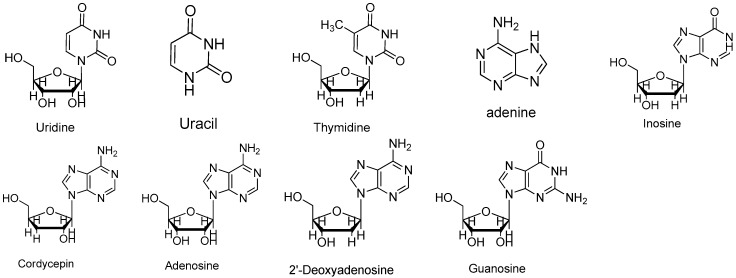
Structures of uridine, uracil, thymidine, adenine, inosine, cordycepin, adenosine, 2′-deoxyadenosine and guanosine.

Standard samples of adenosine, guanosine, uridine, inosine, thymidine, 2-deoxyadenosine adenine, uracil (Sigma-Aldrich, St. Louis, MO, USA), and cordycepin (Chinese Materials Research Centre, Beijing, China) were dissolved in doubly distilled water. The chemical structures of these reference compounds are shown in Figure 6. The determination of the linearity, the correlation coefficient, the reproducibility and the recovery of the nine investigated components were determined. The calibration curve (peak area ratio of the analytes versus the concentration of the analyte, µg/mL) over the studied range of the nine investigated components were calculated. The nucleosides and nucleobases in the samples were identified by comparing the retention times and spiking the *Cordyceps* samples with the standard solutions. The concentrations were calculated using the peak areas of the reference compounds.

### 3.6. HPLC Analysis

The nucleoside analogues and nucleobases were determined by reverse phase HPLC using a Shimadzu Series LC-20 AT high-performance liquid chromatograph (HPLC) (Shimadzu, Kyoto, Japan) equipped with a binary high-pressure pump, and a SPD-20AV UV-Vis detector connected to LC solution software (Shimadzu). Separation was carried out in a pre-packed Diamonsil C18 reverse phase HPLC column (250 mm × 4.6 mm, particle size 5 µm; Dikma Technologies Incorporation, Lake Forest, CA, USA). The absorbance of the eluates was monitored at 260 nm. The mobile phase and elution profile were analysed as described by Xiao et al. [18,19]. The mobile phase was comprised of (A) ultrapure water and (B) methanol. All the solutions were degassed by sonication prior to use. The elution conditions were as follows 0–3 min, isocratic 15% B; 3.0–3.5 min, linear gradient 15–23% B; 3.5–8.5 min, isocratic 23% B; 8.5–9.0 min, linear gradient 23–35% B; and 9.0–15 min, isocratic 35% B, with a final column flush with 100% B for 40 min before reconditioning the column with 15% B isocratic for 30 min, all at 30°C. The flow-rate was 1 mL/min, and the injection volume was 20 µL. All the injections were repeated three times to ensure reproducibility. Nucleosides were identified by comparing the retention time, purity coefficient and spectrum against known chemical standards. The external standard method was used for nucleoside determinations.

### 3.7. ESI/TOF-MS Analysis

For the analysis with the electrospray ionization/time-of-flight mass spectrometer (ESI/TOF-MS) (PerkinElmer, New Orleans, LA, USA), the source voltage, capillary exit voltage, Syringe pump flow rate, drying gas temperature, drying gas rate, spray gas pressure, and evaporator temperature were set to 4.0 kV, 100 V, 10 µL/min, 385 °C, 12 µL/min, 80 psi, and 385 °C, respectively. The samples were analysed in both positive and negative ion modes. The mass-selective detector was manually tuned using a dual nebulizer electrospray source with an internal calibrant consisting of an unknown fluorinated compound delivery system, which introduced a constant flow (10 μL/min) of calibrating solution containing the internal reference masses (*m*/*z* 118.0863, 322.0481) in positive ion mode. Tuning was conducted over the range of *m*/*z* 100–300.

## 4. Conclusions

In this research, the nucleosides content and distribution in five different samples of *O. xuefengensis* (two different natural tissues and three cultured development stages) were systematically analysed by reverse phase HPLC. Currently, nucleosides are believed to be the active components in *Cordyceps*. Seven main nucleosides (cordycepin, adenosine, guanosine, uridine, inosine, thymidine and 2-deoxyadenosine) and two nucleobases (adenineand uracil) were determined in this study. The results showed that the natural fruit body contained the highest amount of adenosine (761.5 ± 56.4 mg/kg), followed by the cultured stroma (721.2 ± 31.8 mg/kg) and cultured mycelium (623.9 ± 15.4 mg/kg), whereas the cadaver of *Phassus nodus* had almost no adenosine content. Inosine was only detected in the natural fruit body and the cadaver of *Phassus nodus*. The adenine, guanosine, uridine and uracil contents were significantly higher in the cultured mycelium than in the natural samples. The natural and cultured *O. xuefengensis*, as well as the reported natural *O. sinensis* by Wang et al. [13], contained roughly the same amounts of adenosine. The cultured *O. xuefengensis* had the higher guanosine concentration than the natural *O. xuefengensis*.

The yield of wild *O. xuefengensis* has been declining in recent years because of its strict growing habitat and excessive harvesting. In addition, the gathering of wild *O. xuefengensis* requires digging out the living root of the medicinal plant *Clerodendrum cyrtophyllum* and cutting open the living root, which leads to damage of the medicinal plant *C. cyrtophyllum*. The cultured *O. xuefengensis* mycelium and stroma may be a promising substitute for natural *O. xuefengensis*, as well as a means forprotecting the natural resource.

## Figures and Tables

**Table 1:** Linear regression data, limit of detection (LOD), limit of quantification (LOQ) for nine analytes by HPLC.

Analyte	Linear Regression Data			LOD (µg/mL)	LOQ (µg/mL)
	Regression Equation	r^2^ (*n* = 9)	Linear Range (µg/mL)		
Uracil	Y = 8.54854× 10^7^x + 19748.4	0.99986	0.5–99.0	0.005	0.015
Uridine	Y = 5.30058 × 10^7^x − 6067.1	0.99997	0.5–149.3	0.010	0.050
Inosine	Y = 4.48713 × 10^7^x + 4348.1	0.99995	0.5–148.5	0.010	0.100
Guanosine	Y = 5.31942 × 10^7^x − 37180.3	0.99902	0.5–147.8	0.010	0.050
Adenine	Y = 9.81843 × 10^7^x − 41387.9	0.99972	0.5–146.3	0.002	0.010
Thymidine	Y = 5.06990 × 10^7^x − 9208.8	0.99995	0.5–147.8	0.015	0.100
Adenosine	Y = 6.75044 × 10^7^x + 13235.0	0.99992	0.5–149.3	0.010	0.050
2-deoxyadenosine	Y = 7.93610 × 10^7^x + 19809.4	0.99991	0.5–151.5	0.002	0.010
Cordycepin	Y = 7.30463 × 10^7^x − 2626.2	0.99991	0.5–150.0	0.010	0.050

**Table 2:** Precision, repeatability, stability and recovery of nine analytes.

Analyte	Precision (RSD, %, *n* = 6)		Stability (RSD, %, *n* = 6)	Recovery (%, *n* = 3)	
	Intraday	Interday		Mean	RSD (%)
Uracil	1.64	2.21	2.05	96.9	5.81
Uridine	1.21	1.94	1.54	100.6	4.78
Inosine	1.00	2.29	2.25	79.4	3.68
Guanosine	2.12	1.75	1.88	80.6	4.71
Adenine	0.96	2.32	2.29	80.6	2.93
Thymidine	1.11	1.60	1.76	102.7	1.32
Adenosine	1.29	2.61	1.71	103.1	4.38
2-deoxyadenosine	1.68	1.49	0.59	101.5	2.75
Cordycepin	1.64	2.80	1.63	90.4	2.88

**Table 3:** Contents (mg/kg) of nine nucleosides and nucleobases in different tissues of *O. xuefengensis.*

Analyte	Natural Fruit Body	Cadaver of Phasus Nodus	Cultured Mycelium	Cultured Stroma	Cultured Coremium
Cordycepin	nd	nd	nd	nd	nd
2-deoxyriboside	nd	nd	62.8 ± 4.9	nd	nd
Adenosine	761.5 ± 56.4aA	nd	623.9 ± 15.4bB	721.2 ± 31.8aAB	582.9 ± 42.6bB
Thymidine	nd	66.8 ± 6.6	nd	nd	nd
Adenine	83.7 ± 8.9cBC	34.8 ± 0.8dC	126.1 ± 9.1bB	155.9 ± 12.2abAB	186.6 ± 16.6aA
Guanosine	513.6 ± 17.9cC	179.7 ± 5.4dD	688.9 ± 14.8aA	626.1 ± 24.8bB	502.1 ± 11.5cC
Inosine	177.6 ± 0.9bB	207.6 ± 2.5aA	nd	nd	nd
Uridine	975.2 ± 41.6bB	506.9 ± 8.1cC	1396.6 ± 49.1aA	869.7 ± 36.0bB	995.2 ± 85.6bB
Uracil	69.2 ± 2.7cC	39.4 ± 0.4eE	87.1 ± 6.9bB	138.1 ± 4.8aA	55.9 ± 1.2dD

a Means ± standard deviation (*n* = 3) with different lowercase and uppercase letters indicate significant difference at the 0.05 level and extremely significant difference at the 0.01 level, by multiple range test, respectively.
